# Genomic of resistance for *Fusarium circinatum* in *Pinus radiata*

**DOI:** 10.1186/1753-6561-5-S7-P28

**Published:** 2011-09-13

**Authors:** Rodrigo Hasbun, Priscila Moraga, Pamela Wachtendorff, Carolina Iturra, Angela Carrasco, Claudio Balocchi, Sofía Valenzuela

**Affiliations:** 1 Genomica Forestal S.A. Edificio Centro de Biotecnologia s/n. Universidad de Concepción. Concepción, Chile; 2 Facultad de Ciencias Forestales. Universidad de Concepción. Concepción, Chile; 3 Genomica Forestal S.A. Edificio Centro de Biotecnologia s/n. Universidad de Concepción. Concepción, Chile; 4 Bioforest S.A. Concepción, Chile

## 

The pitch canker fungus *Fusarium circinatum* was first associated with mortality of *P. radiata* in California during the 1980s. Since then it has been dispersed to several countries of the Northern hemisphere together with South Africa and Chile in the southern hemisphere. In Chile the disease caused mortality of seedlings in *P. radiata* at nursery, and in South Africa the pathogen has already completed its life cycle in plantations. This situation could be replicated in Chile, in the short-term, becoming then a major problem due to that most of the commercial plantations correspond to radiata pine. Research and breeding programs to ensure the availability of resistant trees are essential.

Controlled inoculation trials using different pine species have shown the high susceptibility of *P. radiata*[*1*]. Different families of radiata pine in Australia, New Zealand and Chile have been screened, showing a considerable genetic variation for lesion length and a high heritability depending on the population, suggesting that selection for resistance should result in useful genetic gains [2]. Based on the above findings a molecular approach of pitch canker resistance in *P. radiata* was carried out. This study focuses on the discovery of genome regions associated with resistance and the genes involved in the response mechanism(s), to develop genomic tools for breeding resistance.

The resistance to *F. circinatum* was investigated using a Quantitative Trait Locus (QTL) mapping strategy in a full-sib family of *P. radiata*. This mapping project used a double pseudo testcross approach to map each of the parents with dominant markers. Microsatellite (SSR) and Amplified Fragment Length Polymorphism (AFLP) markers were used to genotype the two parents (XO and XP) and the progeny (86 clones). Both parental maps were joined using the co-dominant SSR and AFLP markers to obtain a saturated genetic map. For each clone (approximately 8 ramets/clone) inoculations were carried out at Bioforest S.A. and lesion lengths were measured 90 days later. Phenotypic data together with genetic map allowed identified and positioned QTLs that control part of the pitch canker resistance in the progeny. Using Windows QTL Cartographer three QTLs were detected for the trait of interest for both parents.
